# The non-gibberellic acid-responsive semi-dwarfing gene *uzu* affects *Fusarium* crown rot resistance in barley

**DOI:** 10.1186/1471-2229-14-22

**Published:** 2014-01-13

**Authors:** Guangdeng Chen, Wei Yan, Yaxi Liu, Yuming Wei, Meixue Zhou, You-Liang Zheng, John M Manners, Chunji Liu

**Affiliations:** 1 CSIRO Plant Industry, 306 Carmody Road, St Lucia QLD 4067, Australia; 2 Triticeae Research Institute, Sichuan Agricultural University, Wenjiang, Chengdu 611130, China; 3 Institute of Ecological and Environmental Sciences, Sichuan Agricultural University, Wenjiang, Chengdu 611130, China; 4 Institute of Food Crops, Jiangsu Academy of Agricultural Science, 50 Zhongling Street, Nanjing 210014, China; 5 Tasmanian Institute of Agriculture and School of Agricultural Science, University of Tasmania, P.O. Box 46, Kings Meadows, Tasmania 7250, Australia; 6 School of Plant Biology, The University of Western Australia, Perth, WA 6009, Australia

**Keywords:** Plant height, Fusarium crown rot, *uzu* gene, Near isogenic lines, DELLA proteins

## Abstract

**Background:**

Studies in *Arabidopsis* show that *DELLA* genes may differentially affect responses to biotrophic and necrophic pathogens. A recent report based on the study of DELLA-producing reduced height (*Rht*) genes in wheat and barley also hypothesized that *DELLA* genes likely increased susceptibility to necrotrophs but increased resistance to biotrophs.

**Results:**

Effects of *uzu*, a non-GA (gibberellic acid)-responsive semi-dwarfing gene, on Fusarium crown rot (FCR) resistance in barley were investigated. Fifteen pairs of near isogenic lines for this gene were generated and assessed under two different temperature regimes. Similar to its impacts on plant height, the semi-dwarfing gene *uzu* also showed larger effects on FCR severity in the high temperature regime when compared with that in the low temperature regime.

**Conclusions:**

Results from this study add to the growing evidence showing that the effects of plant height on *Fusarium* resistances are unlikely related to *DELLA* genes but due to direct or indirect effects of height difference *per se*. The interaction between these two characteristics highlights the importance of understanding relationships between resistance and other traits of agronomic importance as the value of a resistance gene could be compromised if it dramatically affects plant development and morphology.

## Background

One of the critical considerations in cereal breeding is the selection of reduced height (*Rht*) genes. This is because different *Rht* genes do not only affect height differently but may also have different effects on other morphological and agronomic traits of importance [1,2]. It is known that different *Rht* genes can confer dwarfism by different mechanisms. Two of the most widely used *Rht* genes in wheat, *Rht*-*B1b* and *Rht*-*D1b*, are known to encode DELLA proteins which repress GA responsive growth. They are thought to confer dwarfism by producing constitutively active forms of these growth repressors [3]. *Rht8*, however, is not due to defective GA biosynthesis, but to a reduced sensitivity to brassinosteroids [4].

In addition to their roles in plant development, DELLA proteins are believed to differentially affect responses to infections by biotrophic or necrophic pathogens through their influence on the salicylic acid - jasmonic acid balance in *Arabidopsis*[5]. Similar claims were also made recently for wheat and barley by investigating DELLA-producing *Rht* genes in these species [6]. The possibility that *DELLA* genes may play a critical role in disease resistance could drastically impact the efforts of cereal breeding as it could further restrict the options breeders have in exploiting the limited numbers of useful *Rht* genes in each of these crop species. Many previous studies show that *Rht* genes, which may or may not produce DELLA proteins, co-locate with QTL conferring Fusarium head blight (FHB) and Fusarium crown rot (FCR) resistance [7-9]. Considering that accurate assessments for both FCR [10] and FHB [7] are difficult and that resistances to these diseases can be affected by many characteristics including plant height [11,12] and growth rate [13] which often segregate in populations used for QTL mapping, it is not difficult to understand why not all plant height QTL are coincident with those for FHB or FCR susceptibility [14,15].

As opposed to segregating populations where individuals have different genetic backgrounds as well as the disease resistance loci under investigation, differences between the two isolines for a given pair of NILs are often minimal apart from the targeted trait. Thus, assessing genetic effects of a particular gene/trait using NILs can, to a large degree, eliminate the interference of other genes. As a result, NILs are extensively used for analysing effects of a wide array of traits including possible effects of plant height on FHB and FCR. These studies have shown that all *Rht* genes, including those non-GA-responsive ones, affect FHB [11,15-17] and FCR [12,18] resistance in wheat. These results seem to suggest that the effects of plant height on these *Fusarium* diseases are unlikely related to *DELLA* genes. To further clarify if the effects of plant height on *Fusarium* resistance are related to *DELLA* genes, we generated several pairs of NILs for the non-GA-responsive semi-dwarfing gene *uzu* in barley. Responses of these NILs to FCR infection are described in this paper.

## Results

**Figure 1:**
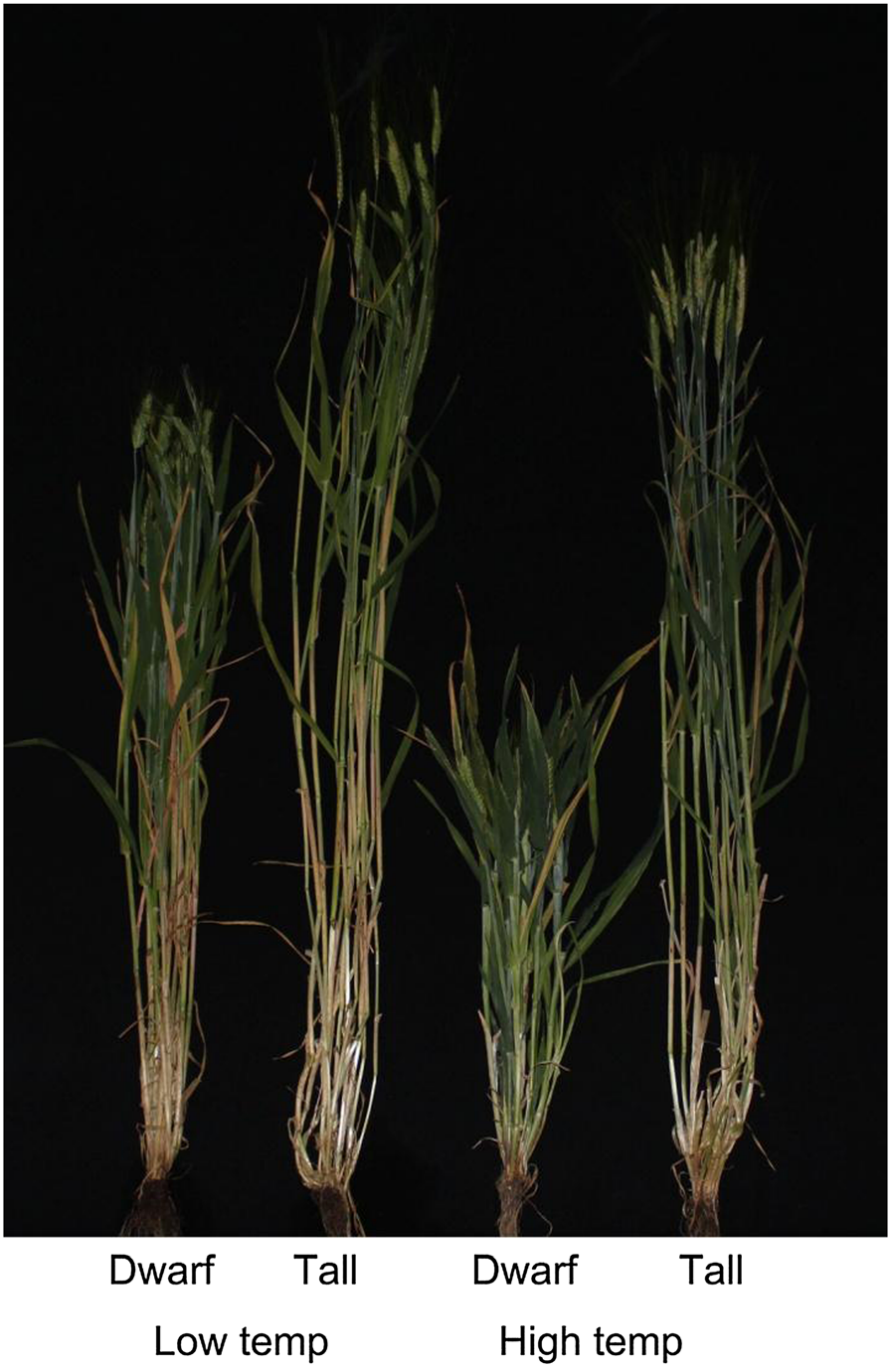
**Differences in plant height between the two isolines for one of the NIL pairs under two temperature regimes**, **showing that the effect of the semi**-**dwarfing gene *uzu *on plant height is more dramatic in the higher temperature regime**.

*Development and assessment of near isogenic lines for the semi*-*dwarfing gene* uzu With the use of the SSR marker HMV33, ten heterozygous plants were identified from the TX9425/Franklin population and five from the TX9425/Gairdner population. Similar to those individuals with homozygous Franklin or Gairdner alleles, all of the 15 heterozygous individuals were characterized as tall plants. However, each of these individuals produced both tall and dwarf progenies. The two isolines for each of the 15 pairs of NILs developed from these heterozygous individuals showed highly significant difference in height under both temperature regimes assessed (Figure 1). In the low temperature regime, the average plant height across the 15 dwarf isolines was 72.0 cm and the 15 tall isolines was 111.3 cm thus the *uzu* gene reduced height by 35.2% on average. In the high temperature regime, the average plant height for the 15 dwarf isolines was 27.8 cm and the 15 tall isolines was 90.4 cm thus the *uzu* reduced height by 69.3% on average. When compared with its effects in the low temperature regime, the average impact of the *uzu* gene on plant height increased in the high temperature regime by 34.1% on average (Table 1).

**Table 1:** **Plant heights of 15 pairs of near isogenic lines for the barley dwarfing gene *uzu *** **under two different temperature regimes**^**#**^

**Background**	**NIL**	**Low temperature**			**High temperature**		
		**Dwarf (cm)**	**Tall (cm)**	**Difference (%)**	**Dwarf (cm)**	**Tall (cm)**	**Difference (%)**
TX9425/ Gairdner	NIL01	69.5 ± 0.9	114.3 ± 1.8	39.2 ± 0.4**	33.2 ± 1.9	84.8 ± 1.2	60.9 ± 2.3**
	NIL02	61.3 ± 0.8	113.3 ± 1.9	45.9 ± 1.4**	17.1 ± 0.3	82.7 ± 2.2	79.3 ± 0.9**
	NIL03	60.8 ± 3.7	104.7 ± 4.9	41.9 ± 2.3**	20.2 ± 0.8	88.8 ± 1.3	77.3 ± 0.9**
	NIL04	70.7 ± 5.4	107.5 ± 2.1	34.3 ± 5.7**	26.8 ± 1.2	89.4 ± 1.5	70.0 ± 1.3**
	NIL05	65.4 ± 2.6	107.3 ± 2.7	39.0 ± 2.5**	25.7 ± 1.1	96.5 ± 2.4	73.4 ± 1.7**
TX9425/ Franklin	NIL06	77.3 ± 1.6	108.5 ± 2.0	28.8 ± 2.3**	27.9 ± 2.5	92.8 ± 2.3	69.9 ± 2.7**
	NIL07	75.0 ± 0.7	119.5 ± 3.7	37.2 ± 1.8**	25.0 ± 3.2	88.1 ± 2.3	71.7 ± 2.8**
	NIL08	75.0 ± 0.6	123.0 ± 1.7	39.1 ± 0.5**	29.7 ± 2.5	98.6 ± 7.3	69.7 ± 4.4**
	NIL09	73.2 ± 3.3	117.3 ± 1.6	37.7 ± 2.2**	24.9 ± 4.1	75.3 ± 2.4	66.9 ± 5.7**
	NIL10	76.1 ± 1.1	114.6 ± 0.7	33.6 ± 0.8**	31.8 ± 1.4	89.3 ± 1.9	64.5 ± 1.7**
	NIL11	76.5 ± 1.6	104.9 ± 3.1	27.1 ± 1.6**	31.0 ± 2.6	96.6 ± 2.6	67.9 ± 2.9**
	NIL12	79.4 ± 0.8	109.5 ± 2.7	27.4 ± 2.4**	33.8 ± 2.5	98.1 ± 2.1	65.6 ± 1.9**
	NIL13	74.1 ± 1.3	112.3 ± 1.7	34.0 ± 1.6**	30.1 ± 1.4	93.9 ± 2.1	68.0 ± 1.2**
	NIL14	78.8 ± 2.1	106.2 ± 2.6	25.7 ± 3.1**	31.1 ± 2.7	89.8 ± 1.8	65.2 ± 3.3**
	NIL15	66.7 ± 0.6	106.6 ± 0.7	37.5 ± 0.8**	28.6 ± 0.6	91.0 ± 2.6	68.6 ± 0.9**

^#^: ‘**’indicate significant level at p < 0.01.

**Figure 2:**
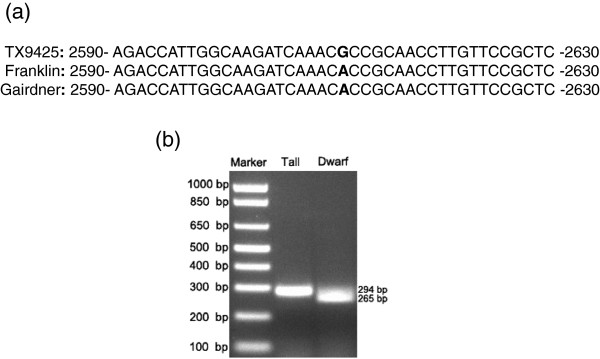
**The characteristic single**-**nucleotide A >G substitution at the position 2612 in the *uzu *gene was present in TX9425 (a); and dCAPS anlysis of the *uzu *allele against one pair of NILs (b).**

Comparisons of genomic DNA sequences among the three parental genotypes used in the development of the NILs confirmed the existence of the *uzu* allele [19] in TX9425. The characteristic single-nucleotide A > G substitution of *uzu* at the position 2612 was the only difference detected along the whole *uzu* gene sequence among the three genotypes (Figure 2a). Analysis of the 15 pairs of NILs using the dCAP markers detected the expected *uzu* allele from each of the 15 dwarf isolines (Figure 2b).

### Differences in Fusarium crown rot severity among the NILs

Difference in disease index (DI) between the two isolines was highly significant for each of the 15 pairs of NILs under both of the temperature regimes assessed. Similar to its impacts on plant height, the semi-dwarfing gene *uzu* also showed larger effects on FCR severity in the high temperature regime than in the low temperature regime. In the low temperature regime, the average DI value across the 15 dwarfing isolines was 44.1 and that for the 15 tall isolines was 61.1, thus the *uzu* gene reduced DI by 27.8% on average. In the high temperature regime, the average DI was 37.9 across the 15 dwarfing isolines and was 55.8 across the 15 tall isolines thus the *uzu* gene reduced DI by 31.7% on average (Table 2).

**Table 2:** **Disease index** (**DI**) **of 15 pairs of near isogenic lines for the barley dwarfing gene *uzu *** **under two different temperature regimes**^#^

**Background**	**NIL**	**Low temperature**			**High temperature**		
		**Dwarf (cm)**	**Tall (cm)**	**Difference (%)**	**Dwarf (cm)**	**Tall (cm)**	**Difference (%)**
TX9425/ Gairdner	NIL01	43.8 ± 2.6	62.5 ± 3.3	30.0 ± 1.0**	41.8 ± 1.0	60.8 ± 2.9	31.2 ± 2.0**
	NIL02	37.8 ± 1.3	56.8 ± 1.0	33.5 ± 1.6**	31.8 ± 1.9	54.0 ± 2.2	41.2 ± 1.3**
	NIL03	38.3 ± 1.5	55.8 ± 1.5	31.4 ± 1.2**	31.8 ± 1.0	51.3 ± 1.7	38.0 ± 1.6**
	NIL04	46.8 ± 2.9	66.5 ± 3.4	29.7 ± 2.2**	41.8 ± 1.3	62.5 ± 1.9	33.2 ± 0.9**
	NIL05	59.0 ± 2.1	87.3 ± 2.2	32.4 ± 0.9**	48.0 ± 1.4	78.3 ± 1.3	38.7 ± 1.3**
TX9425/ Franklin	NIL06	58.8 ± 1.7	72.3 ± 1.7	18.6 ± 0.8**	49.5 ± 1.7	68.2 ± 2.2	27.5 ± 1.0**
	NIL07	40.0 ± 1.4	56.8 ± 1.7	29.5 ± 2.2**	36.0 ± 1.6	52.5 ± 3.1	31.3 ± 3.0**
	NIL08	52.5 ± 1.9	69.3 ± 1.7	24.2 ± 1.1**	40.5 ± 1.3	57.5 ± 1.3	29.5 ± 2.7**
	NIL09	34.0 ± 1.6	48.3 ± 2.2	29.5 ± 1.3**	32.8 ± 1.0	45.3 ± 1.3	27.6 ± 0.9**
	NIL10	36.3 ± 2.1	51.3 ± 3.3	29.2 ± 1.2**	33.3 ± 1.3	48.5 ± 1.9	31.4 ± 1.8**
	NIL11	36.8 ± 2.9	50.8 ± 3.3	27.6 ± 1.6**	33.3 ± 2.0	46.3 ± 1.9	28.1 ± 1.9**
	NIL12	38.3 ± 1.7	53.8 ± 2.6	28.8 ± 1.4**	35.0 ± 1.8	51.3 ± 1.3	31.7 ± 2.3**
	NIL13	34.3 ± 2.2	47.8 ± 3.1	28.3 ± 0.8**	31.5 ± 1.0	44.3 ± 1.7	28.8 ± 1.3**
	NIL14	42.3 ± 1.5	52.3 ± 1.7	19.1 ± 1.4**	36.3 ± 0.9	48.8 ± 1.3	25.6 ± 2.2**
	NIL15	63.0 ± 1.6	84.3 ± 2.2	25.2 ± 0.8**	45.3 ± 2.8	67.0 ± 4.5	32.4 ± 0.9**

^#^:‘**’indicate significant level at p < 0.01.

**Figure 3:**
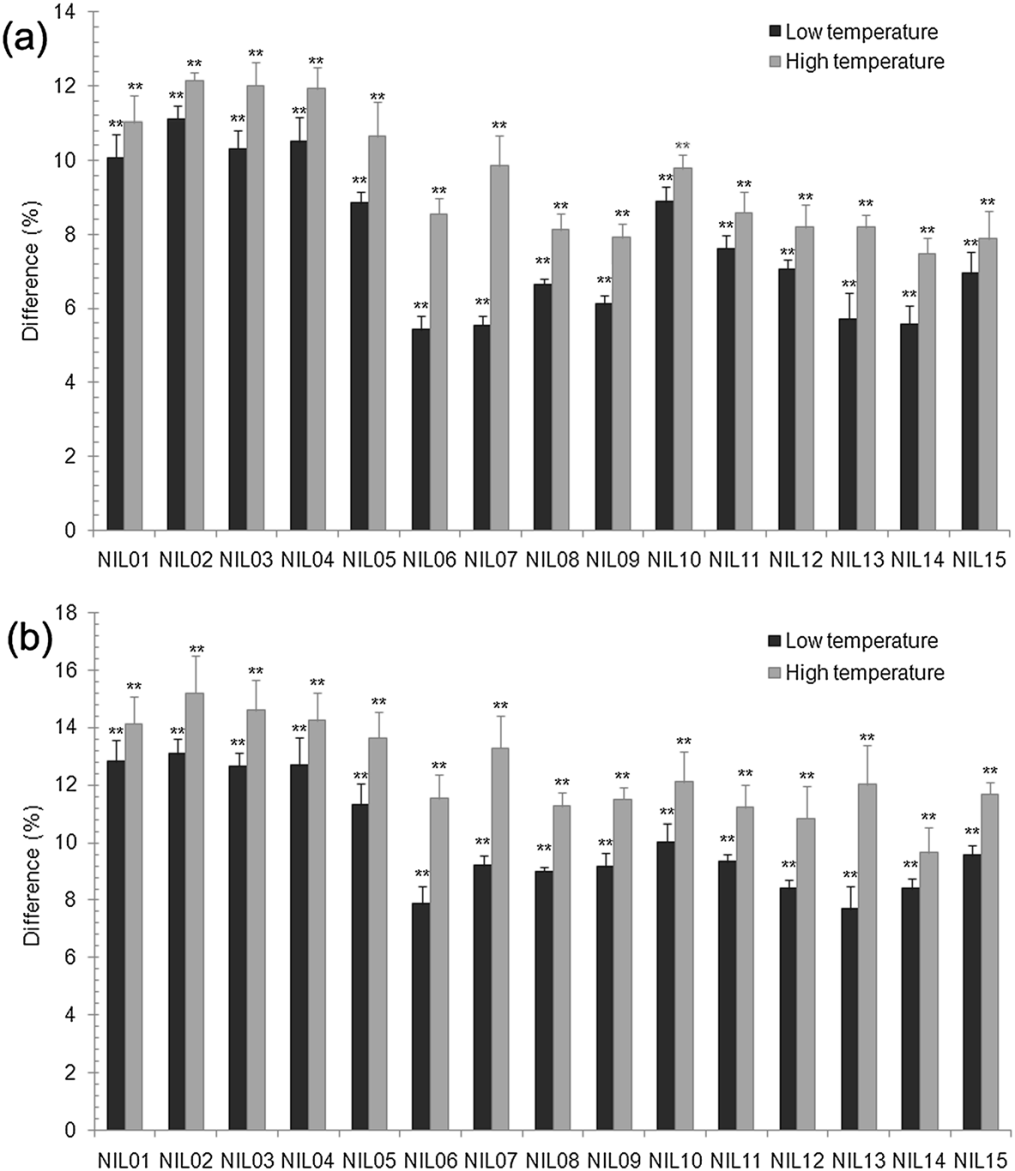
**Differences in *Fusarium *biomass for the 15 pairs of NILs assessed between the two temperature regimes with *Tri5 *(a) or *18 s *(b) as the reference gene.** ‘**’indicate significant level at p < 0.01.

A trend similar to that based on DI values was observed when FCR severity was assessed by estimated biomasses of the *Fusarium* pathogen. The difference between the two isolines was highly significant for each of the 15 pairs of NILs assessed under both of the temperature regimes. The *uzu* allele reduced *Fusarium* biomass by 7.8% and 9.5%, respectively, on average across the 15 pairs of NILs in the low and high temperature regimes when *Tri5* was used as the reference gene (Additional file 1: Figure S1). When *18 s* was used as the reference gene, the *uzu* gene reduced *Fusarium* biomass on average by 10.1% and 12.5%, respectively, across the 15 pairs of NILs in the low and high temperature regimes (Additional file 2: Figure S2). Differences in *Fusarium* biomass between the two isolines for each of the 15 pairs of NILs were significantly larger in the high temperature regime when compared with those in the low temperature regime based on the use of either *Tri5* (Figure 3a) or *18 s* (Figure 3b).

### Correlation between Fusarium crown rot severity and plant height

Plant height was significantly and positively correlated with FCR severity. When FCR severity was measured with DI, the correlation coefficients were 0.64 and 0.73 for data obtained from the low and the high temperature regimes, respectively. When pathogen biomass was used in measuring FCR severity, the correlation coefficients were 0.56 and 0.53 for the low and high temperature regimes, respectively, when *Tri5* was used as the reference gene. The correlation coefficients were 0.67 and 0.57 for the two different temperature regimes, respectively, when *18 s* was used as the reference gene (Table 3).

**Table 3:** **Correlation coefficients between FCR severity and plant height**^#^

**Condition**	Parameter*	**Low temperature**				**High temperature**			
		**PH**	**DI**	***Tri5***	***18 s***	**PH**	**DI**	***Tri5***	***18 s***
Low temperature	PH	1.00							
	DI	0.64**	1.00						
	*Tri5*	0.56*	0.67**	1.00					
	*18 s*	0.67**	0.66**	0.94**	1.00				
High temperature	PH	0.57*	0.38	0.35	0.40	1.00			
	DI	0.72**	0.73**	0.76**	0.75**	0.73**	1.00		
	*Tri5*	0.61*	0.68**	0.88**	0.91**	0.53*	0.80**	1.00	
	*18 s*	0.77**	0.74**	0.79**	0.88**	0.57*	0.81**	0.95**	1.00

‘*’:*PH* = plant height; *DI* = disease index; *Tri5* = *Fusarium* biomass assessed using *Tri5* as the fungal reference gene; *18 s* = *Fusarium* biomass assessed using *18 s* as the fungal reference gene. ^#^: ‘**’ and ‘*’ indicate significant levels at p < 0.01 and p < 0.05, respectively.

## Discussion

The possibility that *DELLA* genes could play a critical role in disease resistance in cereals [6] could seriously restrict options breeders may have in exploiting the limited numbers of useful *Rht* genes in wheat or barley. To further investigate this possibility, we generated 15 pairs of NILs for the semi-dwarfing barley gene *uzu*. These NILs were assessed under two environments between which significant differences in plant height between the two isolines for a given pair of NILs were detected. Highly significant difference in FCR resistance was detected between the two isolines for each of the NIL pairs and the magnitudes of the differences in FCR resistance are associated with the magnitudes of differences in plant height. It is known that the semi-dwarfism conferred by *uzu* is not due to changed sensitivity to GA but to brassinosteroids [19,20]. Thus, the results from this study showed that plant height affects FCR resistance in barley and that the height effects are unlikely related to *DELLA* genes but due to direct or indirect effects of height difference *per se*.

Results from previous studies also show that all *Rht* genes, including those non-GA-responsive ones, affect FCR in wheat [12,18]. There is no ‘cause-and-effect’ that can be implied at this stage yet. However, one of the possible explanations for the reduced FCR severities of the dwarf isolines in both wheat and barley could be their increased cell densities. Considering that FCR is measured by the speed of disease spread within the infected tissues [21,22], the increased cell densities of dwarfing lines [23,24] could form increased physical barriers to pathogen spread within infected tissues. This explanation is supported by the findings that treating plants with exogenous GA increased CR severity as well as seedling lengths in all of the isolines tested, and that the better resistance of the dwarf isolines did not seem to be related to enhanced induction of defense genes [12]. It is known that the growth response of wheat seedlings to exogenous GA application is due to cell expansion not cell division [25,26]. Further evidence supporting the explanation that the increased cell density is likely a contributing factor to the reduced FCR severity is that slow growing genotypes tend to give better CR resistance [13]. It is well known that the genetic control of growth rate is complex and can be conditioned by multiple genes including those for vernalization, photoperiod responses as well as those independent of vernalization and day-length [27]. Nevertheless, it is not unreasonable to speculate that, when compared with those quick growing genotypes, stem elongation of those slow growing genotypes is slower thus their cell densities at stem bases would stay higher for a longer period of time [12].

## Conclusion

By generating and investigating several pairs of NILs for a non-GA-responsive *Rht* gene, we demonstrated in this study that the observed effects of plant height on FCR resistance are not related to *DELLA* genes in barley. These results agree well with previous data on both FCR and FHB in wheat showing overwhelmingly that the observed effects of plant height on resistances to FCR or FHB can be explained by direct or indirect effects of plant height difference *per se*. Results from the interactions between *Rht* loci and *Fusarium* resistance highlight the importance of understanding the possible relationships between resistance and other traits of agronomic importance. It is critical to understand that the value of a resistance gene in breeding programs could be compromised if it dramatically affects plant development and morphology. The effects of *Rht* genes on *Fusarium* diseases described in this paper showed specifically that caution should be taken when considering exploiting any of the numerous FHB or FCR resistance loci co-locating with *Rht* genes in wheat or barley.

## Methods

### Development of near isogenic lines (NILs) for the barley dwarfing gene uzu

NILs for the semi-dwarfing gene *uzu* were developed from two F_2_ populations, TX9425/Franklin and TX9425/Gairdner. The co-dominant SSR marker HVM33 [28], which was most closely linked to the peak of the QTL of dwarfing gene *uzu*, was used to select heterozygous individuals from these populations following the method as described by Chen *et al*. [28]. The selected heterozygous individuals were selfed and the process of selecting heterozygous individuals and selfing was repeated until the F_8_ generation following the ‘fast generation’ procedure described by Zheng *et al*. [29]. Two homozygous lines, one with and the other without the *uzu* allele, were then isolated from the progeny of each of the F_8_ heterozygous plants and were treated as a pair of NILs.

### Confirmation of the uzu allele by molecular cloning

Leaf samples from TX9425, Franklin and Gairdner were collected 4 weeks after germination and frozen immediately in liquid nitrogen and stored in a - 80°C freezer until processing. Frozen samples were ground in a Retsch MM300 Ball mill (Retsch GmbH, Haan, Germany) and approximately 5 mg was used for DNA extraction. DNA was extracted using the QIAGEN plant DNeasy extraction kit (Catalogue number 69103; http://www.qiagen.com) following the manufacturer’s protocol with a final elution volume of 200 μL. PCR was performed in 20 μL reactions containing 1-10 ng genomic DNA, 5 pmol of each primer (Additional file 3: Table S1), 200 μM deoxyribonucleotides, 1.5 mM MgCl_2_, 50 mM KCl, 10 mM Tris-Cl pH 9.0, 0.1% Triton X-100 and 2 units of Taq polymerase. Cycling conditions were: 94°C for 5 min, followed by 35 cycles at 94°C for 45 s, the appropriate annealing temperature (Additional file 3: Table S1) for 45 s, and 72°C for 1 min, with a final extension at 72°C for an additional 15 min. Amplification products were extracted from 1.5% agarose gels using QIAquick Extraction Kit (QIAGEN) and cloned into a TOPO vector (Invitrogen). The transformed competent cells *E. coli* were incubated at 37°C on an LB medium containing Blue-White Select Screening Reagent (Sigma) overnight. Three white colonies for each insert were selected and transferred into a liquid LB medium for plasmid isolation. Plasmid purification was carried out using QIAprep Spin Miniprep Kit (QIAGEN). The inserts were sequenced by Australian Genome Research Facility Ltd.

### Derived cleaved amplified polymorphic sequence (dCAPS) anlysis of the uzu allele in the near isogenic lines (NILs)

To detect the presence or absence of the *uzu* allele in the NILs, dCAPS method was performed as described by Michaels and Amasino [30]. Genomic DNAs from the NILs were amplified using the following primers: forward 5’- GAAATGGAGACCATTGGCAAGATCAAGC -3’ and reverse 5’- CCTTGCCTCCAGATTCTCATCAAC -3’. PCR was performed in 20 μL reactions containing 1-10 ng genomic DNA, 5 pmol of each primer, 200 μM deoxyribonucleotides, 1.5 mM MgCl_2_, 50 mM KCl, 10 mM Tris-Cl pH 9.0, 0.1% Triton X-100 and 2 units of Taq polymerase. Cycling conditions were: 94°C for 5 min, followed by 35 cycles at 94°C for 45 s, 55°C for 45 s and 72°C for 1 min, with a final extension at 72°C for an additional 15 min. PCR products were digested with the restriction enzyme *Hha*I by adding 10 μL of PCR product to 10 μL of the appropriate 1× buffer containing 2 units of *Hha*I. The reactions were incubated at 37°C for 8 h. After digestion, 10 μL of the reaction was analyzed for DNA fragment length on 3% agarose gels.

### Measurement of plant height

Plant heights of the NILs were assessed in four trials in controlled environment cabinets in CSIRO Brisbane laboratories. Each of the trials consisted of two replicates. Three plants, each in a different 2.0 L pot, were used in each of the replicates. For each trial, the pots were arranged in a randomized complete block design. The trials were conducted under two different temperature regimes. Settings for the low temperature regime were 22/16(±1)°C day/night temperature and 65/90(±5)% day/night relative humidity. The settings for the high temperature regime were 28/20(±1)°C day/night temperature and 65/90(±5)% day/night relative humidity. A 14-hour photoperiod with 500 μmol m^-2^ s^-1^ photon flux density at the level of the plant canopy was used for all of the trials. Plant heights were measured using the two tallest tillers for each plant and their averages were used in statistical analysis.

### Fusarium crown rot assessment

The assessment of FCR was conducted using *F. pseudograminearum* isolate CS3096. This isolate is highly aggressive based on an assessment of over 650 isolates collected in field surveys from Queensland and New South Wales [31]. Fungal inoculum was prepared following the method described by Li *et al*. [22]. The spores were harvested and the concentration of macroconidial suspension was adjusted to 1 × 10^6^ spores/ml. Tween 20 was added to the spore suspension to a final concentration of 0.1% vol/vol prior to use for inoculation. Seeds were germinated in Petri dishes on three layers of filter paper saturated with water. The germinated seeds were immersed in the spore suspension for 1 min and two seedlings were planted into a 5 cm square punnet (Rite Grow Kwik Pots, Garden City Plastics, Australia) containing sterilized University of California mix C (50% sand and 50% peat v/v). The punnets were arranged in a randomized block design. To promote FCR development, water-stress was applied during the FCR assessment. Inoculated seedlings were watered only when wilt symptoms appeared.

Four independent trials were conducted in assessing FCR severity. Each of the trials contained two replicates, each replicate with ten seedlings. FCR severity was scored using a ‘0-5’ scale as described by Li *et al*. [22], where ‘0’ representing no symptom and ‘5’ whole seedling completely necrotic. A disease index (DI) was then calculated for each line following the formula of DI = ∑(R_nX_/5 N) × 100, where X is the scale value of each plant, n is the number of plants in the category, and N is the total number of plants assessed for each line.

### Assessment of fungal biomass by quantitative polymerase chain reaction (qPCR)

To analyse fungal biomass, tissue samples were collected 4 weeks after inoculation by removing the lower 2 cm of the seedling base of all 10 seedlings in a replication. The collected samples were immediately immersed in liquid nitrogen and then stored in a - 80°C freezer until processing. Frozen samples were ground in a Retsch MM300 Ball mill (Retsch GmbH, Haan, Germany) and approximately 5 mg was used for DNA extraction. Mycelium of *F. pseudograminearum* and disease free barley tissue in a CEF were similarly ground and used as controls. The ground samples were stored at -20°C until DNA extraction.

DNA was extracted using the QIAGEN plant DNeasy extraction kit (Doncaster, Victoria, Australia) following the manufacturer’s protocol with a final elution volume of 200 μL. Fungal biomass was determined by qPCR as the proportion of barley DNA to fungal DNA with barley actin (AY145451) as the reference gene (forward 5’-GAACAGGAGCTGGAGACTGC-3’ and reverse 5’-ATCATGGATGGCTGGAAGAG-3’). Two reference genes were used for estimating fungal biomass. One was the fungal ribosomal *18 s* gene (forward 5’-GTCCGGCCGGGCCTTTCC-3’ and reverse 5’-AAGTCCTGTTTCCCCGCCACGC-3’) and the other was the *Tri5* gene (forward 5’-GCGCATCGAGAATTTGCA-3’; reverse 5’-TGGCGAGGCTGAGCAAAG-3’). Both *18 s* and *Tri5* have previously been used in estimating *Fusarium* biomass in barley and wheat FCR [32].

The volume used for qPCR amplification was 10 μL containing 5 μL SYBR Green PCR master mix (Applied Biosystems, Scoresy, Victoria, Australia), 1 μL of a 3 mM mix of forward and reverse primers, and 4 μL of sample DNA diluted 1:10 in sterile milliQ water. Following an initial denaturation at 95°C for 10 min, 45 cycles each of 15 s denaturing at 95°C and 1 min annealing/elongation step at 60°C were used in qPCR. A final denaturation step at 95°C for 2 min, annealing at 60°C, and denaturing at 95°C for 15 s was added to determine the melting temperature of the amplified product in the form of a dissociation curve. qPCR was performed on 384 well plates, each DNA extract (biological replication) was analysed in three replicated wells for each pair of primers. The average value from the three replicated wells was used as data for each biological replication. Fusarium DNA relative to barley DNA was calculated as an estimate of relative biomass using the following equation [33]:

$$\mathrm{Relative}\;\mathrm{biomass}=\frac{Ef_{\mathrm{Fungal}}^{-\mathrm{Ct}}}{Ef_{\mathrm{Plant}}^{‒\mathrm{Ct}}}$$

where Ef is PCR amplification efficiency determined using LINREGPCR 7.5 [34] and Ct is the crossing threshold.

### Statistical analyses

Statistical analyses were performed using the SPSS statistics 17.0 for Windows (SPSS Inc., Chicago, IL). For each trial, the following mixed-effect model was used: Yij = μ + *ri* + *gj* + *wij*. Where: Y*ij* = trait value on the *j*th genotype in the *i*th replication; μ = general mean; *ri* = effect due to *i*th replication; *gj* = effect due to the *j*th genotype; *wij* = error or genotype by replication interaction, where genotype was treated as a fixed effect and that of replicate as random. Pearson correlation coefficients were estimated between plant height, DI value and *Fusarium* biomass.

## Competing interests

The authors declare that they have no competing interests.

## Authors’ contributions

CL, JMM, Y-LZ, MZ and YW conceived and designed the experiments. GC and CL developed the near isogenic lines. GC and WY contributed to the Fusarium crown rot assessment. GC and YL performed the gene cloning, dCAPS and qPCR analysis. GC, YL and CL analyzed the data. CL, GC, JMM, Y-LZ, MZ and YW wrote the manuscript. All the authors read and approved the final manuscript.

## Supplementary Material

Additional file 1: Figure S1 Relative biomass of *Fusarium* between the two isolines for each of the 15 pairs of NILs under the low - (a) and high - temperature (b) regimes with *Tri5* as the reference gene. Click here for file

Additional file 2: Figure S2 Relative biomass of *Fusarium* between the two isolines for each of the 15 pairs of NILs under the low - (a) and high - temperature (b) regimes with *18 s* as the reference gene. Click here for file

Additional file 3: Table S1 Primer sequences used for *uzu* allele detection. Click here for file
