# Supplementary material for: The non-gibberellic acid-responsive semi-dwarfing gene uzu affects Fusarium crown rot resistance in barley
Source: BMC Plant Biol. 2014 Jan 13;14:22. doi: 10.1186/1471-2229-14-22 (PMC3898025; doi:10.1186/1471-2229-14-22)
Supplement: Additional file 1: Figure S1 — Relative biomass of Fusarium between the two isolines for each of the 15 pairs of NILs under the low - (a) and high - temperature (b) regimes with Tri5 as the reference gene. [file 1471-2229-14-22-S1.docx]

**Supplementary Table S1.** Primer sequences used for *uzu* allele detection

| Fragment of uzu gene | Forward primer (5’-3’) | Reverse primer (5’-3’) | Annealing temperature(℃) | Reference# |
| --- | --- | --- | --- | --- |
| uzu1 | CGCTTCTCGCATGGTCTC | CAGCGAAGGTCGGCATCT | 49.5 | Developed in this study |
| uzu2 | CTCGACTTGTCCAGCAACAA | GTTGGGATCTTGGCAGAGG | 57.5 | Gruszka *et al*., 2011 |
| uzu3 | CGACCTCAGCTCCAACAACT | TCCTTGTGAAGTTGCACAGC | 55 | Gruszka *et al*., 2011 |
| uzu4 | CTGAGCAGCCAGTGTCGT | TACTTGCCTCGTCATTCTTCT | 55 | Developed in this study |
| uzu5 | GGAGGCAGAAGAATGACGAG | CAGCAACACAACACCGTAGC | 57.5 | Gruszka *et al*., 2011 |
| uzu6 | GGTATGTGCCACCGGAGTA | AGCTTGCGTGGGAACCTCA | 55 | Gruszka *et al*., 2011 |

# Gruszka D, Szarejko I, Maluszynski M: **New allele of *HvBR1* gene encoding brassinosteroid receptor in barley**. *J Appl Genet* 2011, **63:**257-268.
