# Supplementary figures and images for: The non-gibberellic acid-responsive semi-dwarfing gene uzu affects Fusarium crown rot resistance in barley
Source: BMC Plant Biol. 2014 Jan 13;14:22. doi: 10.1186/1471-2229-14-22 (PMC3898025; doi:10.1186/1471-2229-14-22)

## Slide 1
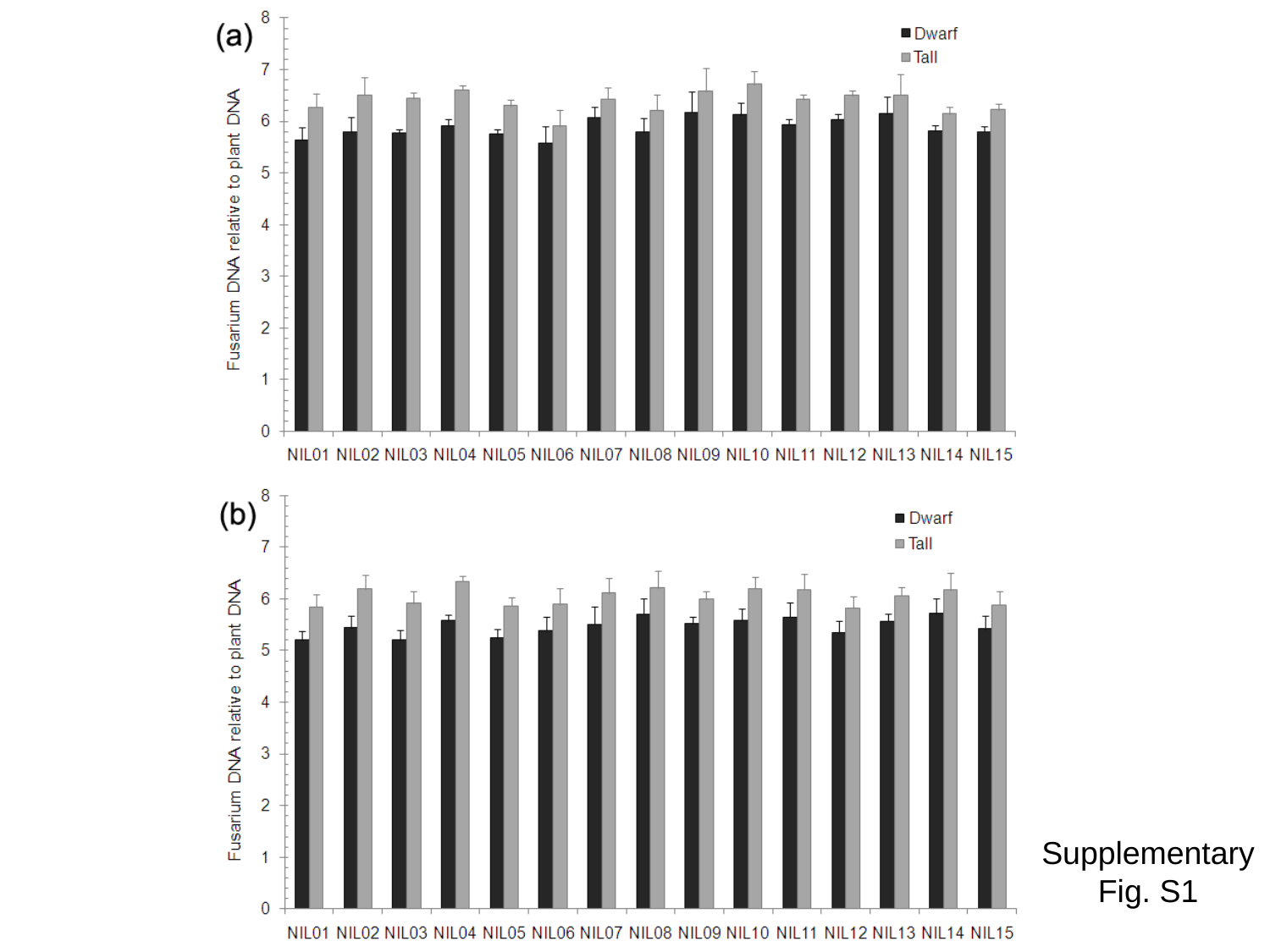

Supplementary
Fig. S1

Supplement: Additional file 2: Figure S2 — Relative biomass of Fusarium between the two isolines for each of the 15 pairs of NILs under the low - (a) and high - temperature (b) regimes with 18 s as the reference gene. [file 1471-2229-14-22-S2.pptx]

## Slide 1
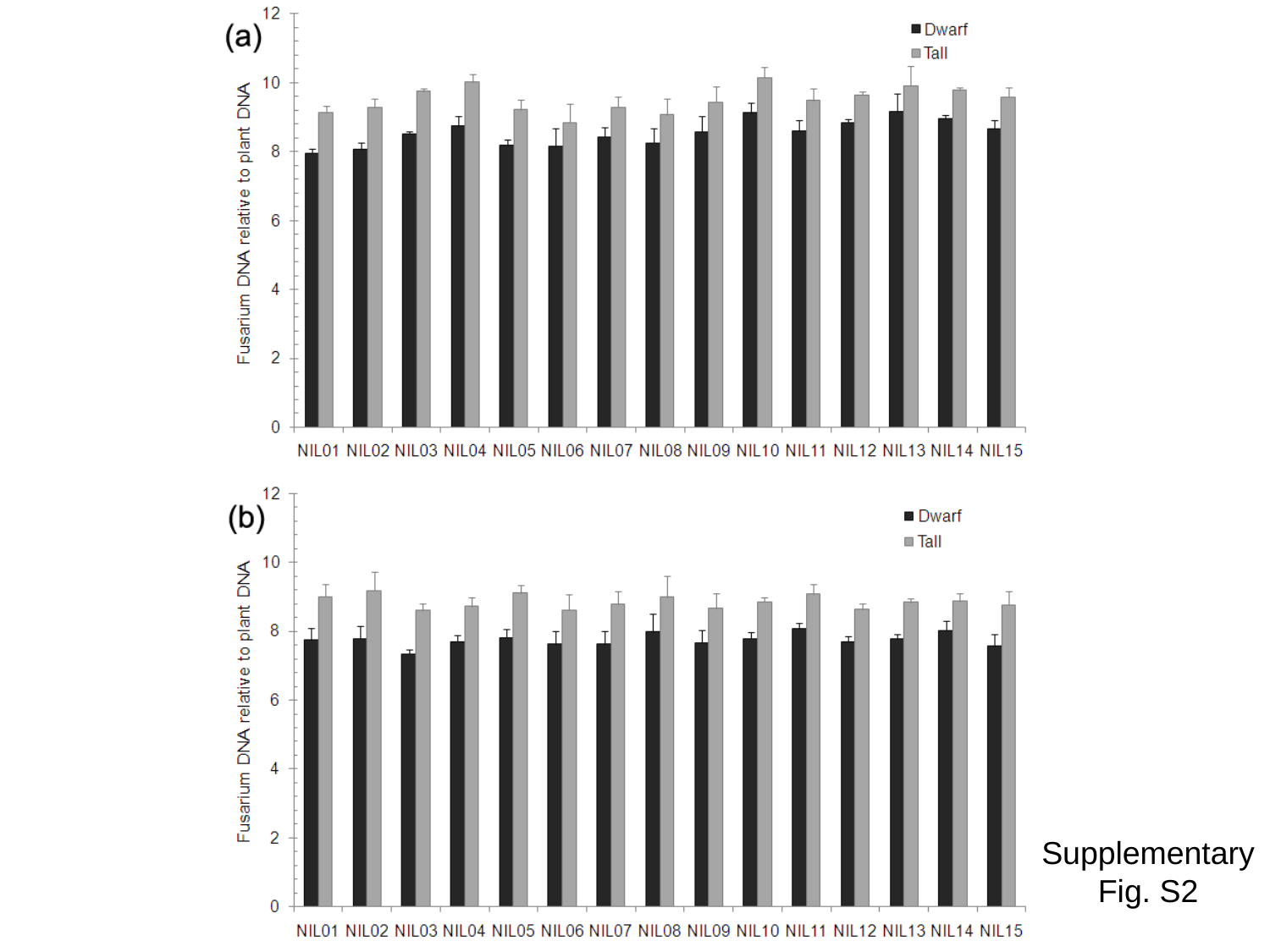

Supplementary
Fig. S2

Supplement: Additional file 3: Table S1 — Primer sequences used for uzu allele detection. [file 1471-2229-14-22-S3.pptx]
